# Repeated tube exposure due to masked herpes zoster ophthalmicus after Ahmed valve implantation in an eye with neovascular glaucoma: A case report

**DOI:** 10.1097/MD.0000000000041030

**Published:** 2024-12-27

**Authors:** Hirotaka Tanabe, Shunsuke Nakakura, Yoshie Shimizu, Sachiko Maruoka, Tomohiro Shojo

**Affiliations:** a Department of Ophthalmology, Yokkaichi Digestive Disease Center, Yokkaichi, Japan; b Department of Ophthalmology, Tsukazaki Hospital, Himeji, Japan.

**Keywords:** Ahmed valve, herpes zoster ophthalmicus, neovascular glaucoma, tube exposure

## Abstract

**Rationale:**

Herpes zoster ophthalmicus (HZO) occurs after the reactivation of latent varicella-zoster virus (VZV) present within the sensory spinal or cerebral ganglia and sometimes causes ocular inflammatory diseases, including neovascular glaucoma (NVG), which is one of the most devastating sequelae of virus-associated uveitis. In NVG, the synechial angle ultimately closes due to the contraction of proliferating myofibroblasts, which often requires glaucoma filtration surgery, including Ahmed glaucoma drainage device implantation, to maintain optimum intraocular pressure (IOP). Although tube exposure from the conjunctiva is a complication of glaucoma implant surgery, a case in which a glaucoma tube implant was repeatedly exposed due to masked HZO in a postoperative eye with NVG has not been reported.

**Patient concerns:**

A 72-year-old male with NVG in his left eye ultimately underwent Ahmed glaucoma valve implantation with sulcus fixation for uncontrolled IOP. Before surgery, acyclovir ophthalmic ointment had been applied to treat a suspected VZV infection, but the regimen was discontinued because an aqueous humor sample tested negative for VZV according to polymerase chain reaction. Postoperatively, the patient’s IOP dramatically decreased without any need for glaucoma eye drops. However, the tube was exposed when the overlying conjunctiva and allogeneic scleral grafts disintegrated.

**Diagnoses:**

Although the tube was covered with different types of tissues, including an allogeneic corneal graft and an autogenous conjunctival graft from the same eye, the grafts completely disintegrated again. Covering the tube with another allogeneic corneal graft and an autogenous free conjunctival graft from the contralateral eye also failed, with gradual disintegration of these tissues. During the observation period, severe HZO with Hutchinson sign and clearly demarcated pseudodendritic corneal ulceration were noted.

**Interventions:**

After thorough consideration, we cut and removed the tube to avoid possible scleral perforation due to prolonged inflammation.

**Outcomes:**

Fortunately, the patient’s IOP was controlled by resuming the continuous daily application of antiviral ointment and repeatedly injecting anti-vascular endothelial growth factor for rubeosis regularly afterward.

**Lessons:**

The possibility of VZV reactivation should always be considered in cases of tube exposure due to an unknown cause after Ahmed valve implantation in an eye with NVG.

## 1. Introduction

Herpes zoster ophthalmicus (HZO) occurs after reactivation of latent varicella-zoster virus (VZV), which is present within the sensory spinal or cerebral ganglia and features a unilateral painful rash in ≥1 dermatome of the trigeminal nerve, shared by the eye and ocular adnexa.^[1]^ Although many parents or children may not remember or may not have recognized their prior cases of varicella in the pre-vaccination era, 95% of the population has serological evidence of prior VZV infection.^[2]^ VZV is sometimes responsible for ocular inflammatory diseases, including neovascular glaucoma (NVG), which is one of the most devastating sequelae of virus-associated uveitis. Previous reports have demonstrated that herpetic virus–associated anterior uveitis (VAU), which includes cytomegalovirus, herpes simplex virus (HSV), and VZV, is the most common VAU and accounts for 5% to 10% of all uveitis cases seen at tertiary referral centers,^[3–6]^ and secondary glaucoma develops in 10% to 40% of patients with VAU during the course of the disease.^[7–13]^ Hoeksema et al^[14]^ reported that 19% of patients with VAU (VZV and HSV) needed surgical intervention, consisting mainly of the implantation of a Baerveldt glaucoma drainage device (11/14; 79%) to control individually elevated intraocular pressure (IOP) levels. Pohlmann et al^[13]^ demonstrated that 10% of patients with VAU needed glaucoma surgery, which includes minimally invasive glaucoma surgery involving trabectome surgery and iStent injection implantation as first-line treatment and filtrating glaucoma surgery, such as trabeculectomy, as second-line therapy; although minimally invasive glaucoma surgery showed a reliable IOP reduction in the short term, follow-up surgery was required in 37% of patients. HSV and VZV are clinically similar, but elevated IOP is more common in patients with VZV than in patients with HSV, and vitritis is more prominent in patients with VZV than in patients with HSV.^[13]^

Tube exposure from the conjunctiva is one of the complications of glaucoma implant surgery. A variety of materials have been used as patch materials, including preserved sclera, cornea, and autologous or processed sclera, and prospective studies on tube exposure have reported an incidence of 1% to 5% over a 5-year follow-up.^[15–17]^ However, a high incidence of 5.8% to 8.3% has been reported in retrospective studies, regardless of the patch material.^[18–21]^ The obvious cause of tube exposure is currently unknown but has been attributed to mechanical irritation, immune reactions to foreign materials, and other factors.^[22,23]^ Various risk factors for tube exposure, including smoking, pseudoexfoliation glaucoma, dry eye syndrome, mitomycin C use, female sex, White race, younger age, inflammation, Hispanic ethnicity, NVG, previous trabeculectomy, and concurrent surgery, have been suggested.^[19,20,24,25]^ Although tube exposure from the conjunctiva is a common problem after glaucoma implant surgery, cases of glaucoma tube exposure due to the recurrence of latent VZV have not been reported. In this report, we present a case in which repeated exposure of a glaucoma tube implant due to masked HZO was observed in a postoperative eye with NVG.

## 2. Case report

**Figure 1.**
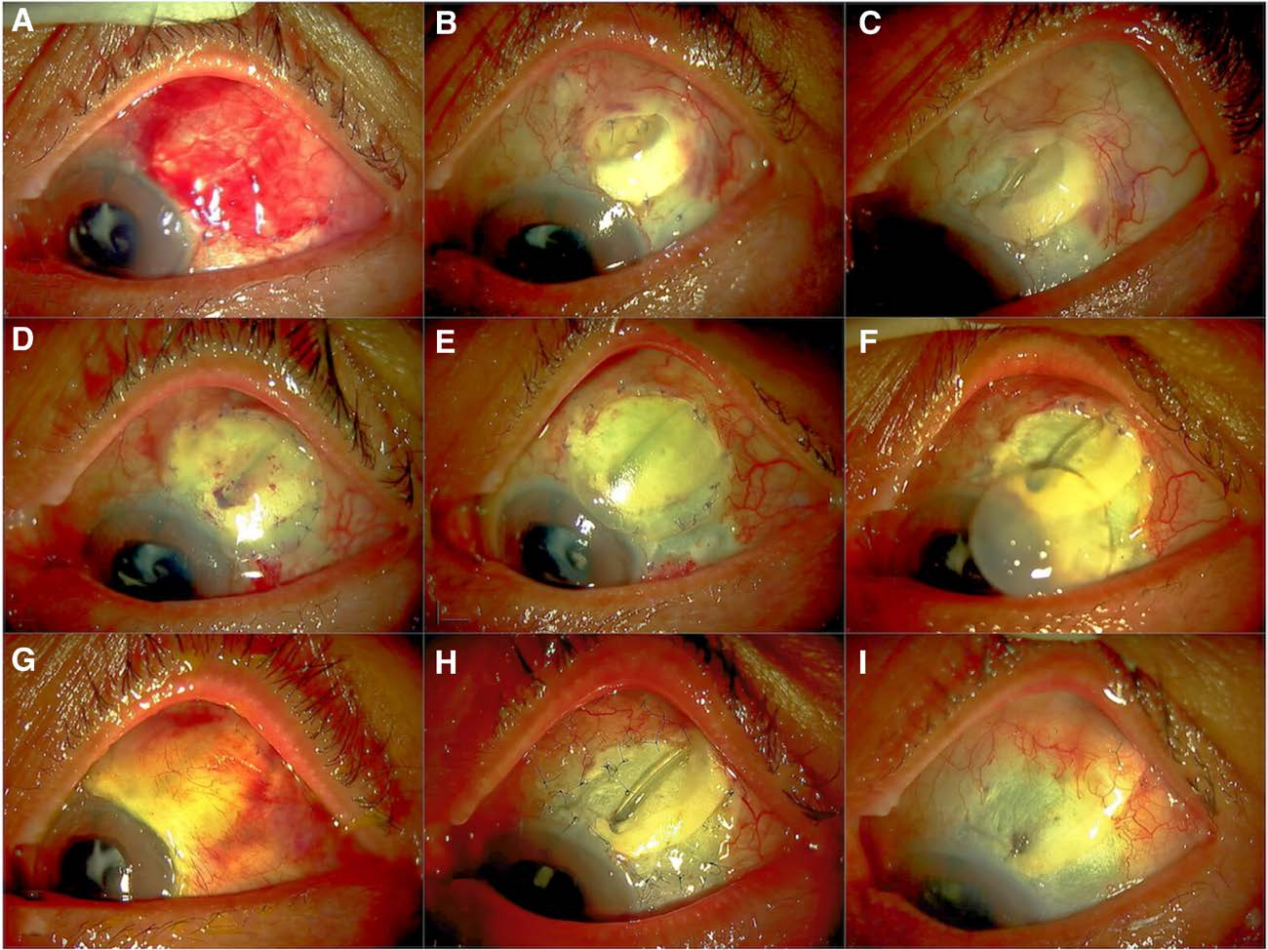
Repeated Ahmed tube exposure after Ahmed valve implantation in an eye with neovascular glaucoma. Following Ahmed glaucoma valve implantation on August 2, 2020 (A), the conjunctiva and allogeneic scleral graft covering the implant gradually deteriorated (B), leading to exposure of the Ahmed tube (C). Despite covering the tube again with an allogeneic corneal graft and an autogenous conjunctival graft from the same eye on December 5, 2020 (D), these grafts also degraded completely (E and F). Subsequently, another allogeneic corneal graft and an autogenous free conjunctival graft from the opposite eye were used to cover the tube on December 25, 2020 (G), resulting in the gradual but eventual complete disintegration of these tissues (H). Finally, we cut and removed the tube on February 27, 2021 (I).

A 72-year-old male with NVG in his left eye was referred to Tsukazaki Hospital for uveitis treatment on July 27, 2019. He had a history of surgical interventions, including vitrectomy and simultaneous cataract surgery for retinal detachment in both eyes. His left eye had been diagnosed with amblyopia. The corrected visual acuity of his left eye was 20/200, and the IOP was 28 mm Hg with the maximum glaucoma eye drop dose. Examination revealed old panretinal photocoagulation scars on the retina of both eyes from previous retinal detachment treatment. First, we controlled the IOP by prescribing the full dose of glaucoma eye drops, a steroidal eye drop, or acyclovir ophthalmic ointment for suspected VZV infection without decisive signs. Although anti-vascular endothelial growth factor (VEGF) intravitreal injections were also performed on a sporadic basis to stop NVG progression, the patient’s IOP gradually increased due to exacerbated angle closure caused by neovascular progression. Unfortunately, laser iridotomy was ineffective. After careful observations, we ultimately performed Ahmed glaucoma valve implantation with sulcus fixation for uncontrolled IOP in his left eye on August 2, 2020 (Fig. 1A). After these procedures, we stopped the acyclovir ophthalmic ointment treatment, which had been continued to treat a suspected VZV infection because an aqueous humor sample tested negative for VZV according to polymerase chain reaction. Postoperatively, the patient’s IOP dramatically decreased without any need for glaucoma eye drops. However, the overlying conjunctiva and allogeneic scleral graft gradually disintegrated (Fig. 1B), and the Ahmed tube was ultimately exposed (Fig. 1C). Although the tube was covered with different types of tissues, that is, an allogeneic corneal graft and an autogenous conjunctival graft from the same eye on December 5, 2020 (Fig. 1D), the grafts completely disintegrated again (Fig. 1E and 1F). The tube was covered with another allogeneic corneal graft and an autogenous free conjunctival graft from the contralateral eye on December 25, 2020 (Fig. 1G), which also failed with the gradual but ultimately complete disintegration of these tissues (Fig. 1H).

**Figure 2.**
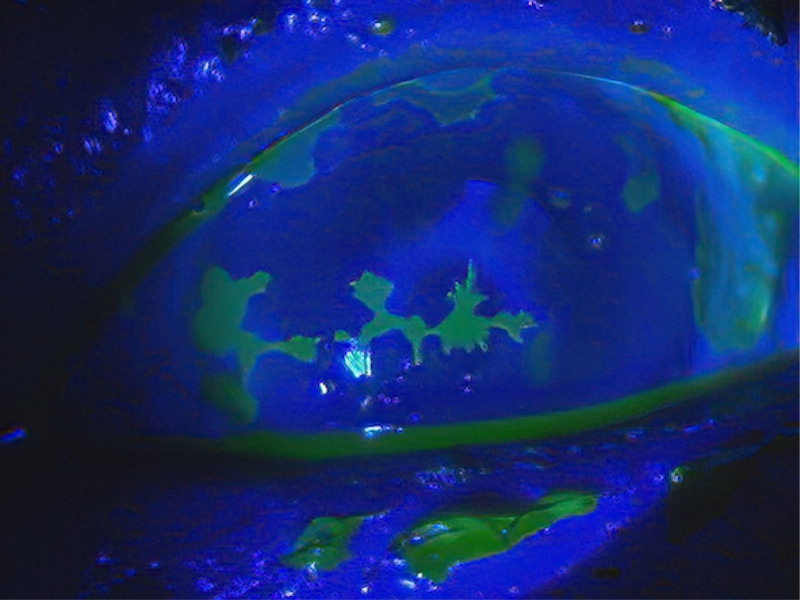
Demarcated pseudodendritic corneal ulceration with herpes zoster ophthalmicus in an eye with neovascular glaucoma, which was photographed on January 28, 2021. Characteristic varicella-zoster virus pseudodendrite findings were observed; they were slightly elevated, broader, and polymorphous, with less regular branching and few terminal dilatations.

During the observation period, severe HZO with Hutchinson sign and clearly demarcated pseudodendritic corneal ulceration (Fig. 2) were noted on January 28, 2021. After thorough consideration, we cut and removed the tube on February 27, 2021 (Fig. 1I) to avoid possible scleral perforation due to prolonged inflammation. Fortunately, the patient’s IOP was controlled by resuming the continuous daily application of antiviral ointment, reducing the use of steroidal eye drops, and repeatedly injecting anti-VEGF for rubeosis regularly afterward.

## 3. Discussion

In our observation of a patient with NVG, surgical intervention repeatedly failed due to masked VZV infection with late-onset decisive signs of severe HZO with Hutchinson sign and clearly demarcated pseudodendritic corneal ulceration (Fig. 2). We finally achieved stable IOP by cutting and removing the exposed Ahmed glaucoma tube and resuming acyclovir ophthalmic ointment treatment and anti-VEGF intravitreal injections regularly. Liesegang^[26]^ reported that 65% of patients with HZO had corneal involvement, 34.4% had ocular symptoms (pain, tearing, and foreign-body sensation) before skin eruption, and 85.2% had dermatologic evidence of involvement of the nasociliary division of the ophthalmic nerve. However, no patients had isolated involvement of the nasociliary division according to a retrospective chart review at the Mayo Clinic.^[26]^ In our patient, the HZO skin lesion did not appear until the very late stage. Treatment with acyclovir ophthalmic ointment before a negative polymerase chain reaction result for VZV in an aqueous humor sample might have delayed the HZO appearance. Liesegang^[1]^ reported that in HMO, the surface of the globe may exhibit episcleritis or deeper scleritis in the early stages of the disease that may become persistent or appear in a delayed fashion. Although the patient’s IOP dramatically decreased without any need for glaucoma eye drops after Ahmed glaucoma valve implantation, the Ahmed tube was repeatedly exposed due to prolonged inflammation, which caused disintegration of the overlying conjunctiva and allogeneic scleral graft after the first operation, another allogeneic scleral graft and an autogenous free conjunctival graft from the same eye after the second operation, and an allogeneic corneal graft and an autogenous free conjunctival graft from the contralateral eye after the third operation. We were not sure if the fourth trial, which involved covering the tube with another graft while resuming the acyclovir ophthalmic ointment treatment, would be successful. Recently, Nakakura et al^[27]^ reported a novel surgical technique of covering the tube with an inverted sclera to avoid tube erosion effectively, which might have been a promising first-line therapy in this complicated case. However, considering the risk of scleral rupture due to scleral thinning caused by chronic inflammation, we decided to cut and remove the tube. We removed the inserted part of the Ahmed tube and cut it at the most distant site on the outer side of the eye. Even though we closed the opening of the sclera by suturing, we have regained control of the patient’s IOP by resuming the continuous daily application of antiviral ointment and repeatedly injecting anti-VEGF for NVG rubeosis regularly afterward.

## 4. Conclusion

Even when no decisive signs are observed, the possibility of VZV reactivation should always be considered if a postoperative glaucoma implant is exposed by an unknown cause in an eye with NVG.

## Acknowledgments

The authors thank the staff of Tsukazaki Hospital who were involved in this patient’s care. American Journal Experts edited the language of the manuscript.

## Author contributions

**Conceptualization:** Hirotaka Tanabe, Shunsuke Nakakura

**Investigation:** Hirotaka Tanabe

**Project administration:** Hirotaka Tanabe

**Resources:** Hirotaka Tanabe, Shunsuke Nakakura, Tomohiro Shojo

**Supervision:** Hirotaka Tanabe, Shunsuke Nakakura, Yoshie Shimizu, Sachiko Maruoka

**Validation:** Hirotaka Tanabe, Shunsuke Nakakura

**Visualization:** Hirotaka Tanabe

**Writing – original draft:** Hirotaka Tanabe

**Writing – review & editing:** Hirotaka Tanabe, Shunsuke Nakakura
